# Hepatitis C virus core protein targets 4E-BP1 expression and phosphorylation and potentiates Myc-induced liver carcinogenesis in transgenic mice

**DOI:** 10.18632/oncotarget.17280

**Published:** 2017-04-20

**Authors:** Cosette Abdallah, Charlène Lejamtel, Nassima Benzoubir, Serena Battaglia, Nazha Sidahmed-Adrar, Christophe Desterke, Matthieu Lemasson, Arielle R. Rosenberg, Didier Samuel, Christian Bréchot, Delphine Pflieger, François Le Naour, Marie-Françoise Bourgeade

**Affiliations:** ^1^ Inserm, Unité 1193, Villejuif, F-94800, France; ^2^ University Paris-Sud, UMR-S 1193, Villejuif, F-94800, France; ^3^ Inserm, UMS33, Villejuif, F-94800, France; ^4^ Univ Paris-Sud, UMS33, Villejuif, F-94800, France; ^5^ AP-HP Hôpital Paul Brousse, Centre Hépatobiliaire, Villejuif, F-94800, France; ^6^ University Paris-Sud, UMS33, Villejuif, F-94800, France; ^7^ CNRS, LAMBE, UMR 8587, Université d’Evry Val d’Essonne, Evry, F-91000, France; ^8^ Université Evry Val d’Essonne (UEVE), LAMBE, Evry, F-91000, France; ^9^ DHU Hepatinov, Villejuif, F-94800, France; ^10^ Université Paris Descartes, EA4474, Paris, F-75014, France; ^11^ Present address: EDyP, Laboratoire Biologie à Grande Echelle, Institut de Biosciences et Biotechnologies de Grenoble, CEA, Grenoble, F-38054, France

**Keywords:** HCV core, 4E-BP1 phosphorylation, hepatocellular carcinoma, phosphoproteomics, SILAC

## Abstract

Hepatitis C virus (HCV) is a leading cause of liver diseases including the development of hepatocellular carcinoma (HCC). Particularly, core protein has been involved in HCV-related liver pathologies. However, the impact of HCV core on signaling pathways supporting the genesis of HCC remains largely elusive. To decipher the host cell signaling pathways involved in the oncogenic potential of HCV core, a global quantitative phosphoproteomic approach was carried out. This study shed light on novel differentially phosphorylated proteins, in particular several components involved in translation. Among the eukaryotic initiation factors that govern the translational machinery, 4E-BP1 represents a master regulator of protein synthesis that is associated with the development and progression of cancers due to its ability to increase protein expression of oncogenic pathways. Enhanced levels of 4E-BP1 in non-modified and phosphorylated forms were validated in human hepatoma cells and in mouse primary hepatocytes expressing HCV core, in the livers of HCV core transgenic mice as well as in HCV-infected human primary hepatocytes. The contribution of HCV core in carcinogenesis and the status of 4E-BP1 expression and phosphorylation were studied in HCV core/Myc double transgenic mice. HCV core increased the levels of 4E-BP1 expression and phosphorylation and significantly accelerated the onset of Myc-induced tumorigenesis in these double transgenic mice. These results reveal a novel function of HCV core in liver carcinogenesis potentiation. They position 4E-BP1 as a tumor-specific target of HCV core and support the involvement of the 4E-BP1/eIF4E axis in hepatocarcinogenesis.

## INTRODUCTION

Hepatocellular carcinoma (HCC) is the sixth most prevalent cancer and the second most common cause of cancer-related deaths worldwide. Its incidence is still increasing [1]. Hepatitis C Virus (HCV) infection is one of the major etiologies of HCC. Major progress in HCV therapy now leads to envisage the eradication of HCV infection in a near future. However, these new treatments do not directly interfere with liver carcinogenesis and therefore many patients already at the stage of HCV-induced cirrhosis will still develop HCC. Furthermore, there is accumulating evidence that there are common pathways and related mechanisms that likely account for viral and non-viral pathogenesis of cancers [2]. Thus, elucidating the pathways implicated in the progression of HCV-induced liver disease and HCC is still an unmet need for developing novel therapeutic approaches.

The involvement of viral and host factors in the progression of HCV-induced liver disease is intricate. It has been suggested that HCV-encoded proteins are directly involved in the tumorigenic process through interaction with a number of host factors and signaling pathways. Extensive studies have suggested that HCV core protein binds several cellular proteins and plays a major role in HCV-induced liver pathologies by modulating multiple cellular processes. Among them, we have reported that HCV core protein inhibits the canonic TGF-β signaling pathway [3] and shifts its biological responses from tumor suppression to tumor progression by decreasing hepatocyte apoptosis and increasing epithelial mesenchymal transition (EMT) [4]. Strikingly, an HCV core sequence isolated from an HCC nodule was more potent to inhibit TGF-β signaling than a core sequence isolated from the adjacent non-tumor tissue, highlighting the ability of HCV core variants to display different biological host responses [4].

Several studies conducted on transgenic animal models have reported that HCV core expression led to either no particular phenotype [5], steatosis [6, 7], insulin resistance [8, 9], modulation of apoptosis [10, 11] or even HCC development [12]. This is consistent with the notion that these various observations might be correlated with HCV genotype, mouse genetic background [13], or HCV core levels related to the chosen promoter as well as with core sequences used in the different studies. The establishment of transgenic mouse models expressing core sequences isolated from tumor or non-tumor nodules of the same patient would be an invaluable tool to investigate the pathological potential of these HCV core variants and the molecular basis downstream.

To decipher the molecular events involved in the oncogenic potential of HCV core, a global quantitative phosphoproteomic approach of HCV core expressing cells represents a highly valuable tool to investigate the activation or repression of host cell signaling transduction pathways. Indeed, no data are available on the global profile of the HCV core regulated phosphoproteome. We here report the identification of a panel of proteins phosphorylated in HCV core expressing cells involved in metabolism, cell death, protein transport and cellular component organization as well as in the regulation of translation. In particular, we demonstrated that HCV core activates a pathway increasing the phosphorylated level of the key translation factor eukaryotic translation initiation factor 4E-binding protein 1 (4E-BP1) in primary human and mouse hepatocytes as well as in HCV core transgenic mouse livers. We also report that HCV core is able to potentiate Myc-induced tumor development in double transgenic mice and 4E-BP1 might be responsible for this acceleration of tumor progression.

## RESULTS

### Quantitative phosphoproteomic analysis of hepatoma cell line expressing HCV core protein

**Figure 1:**
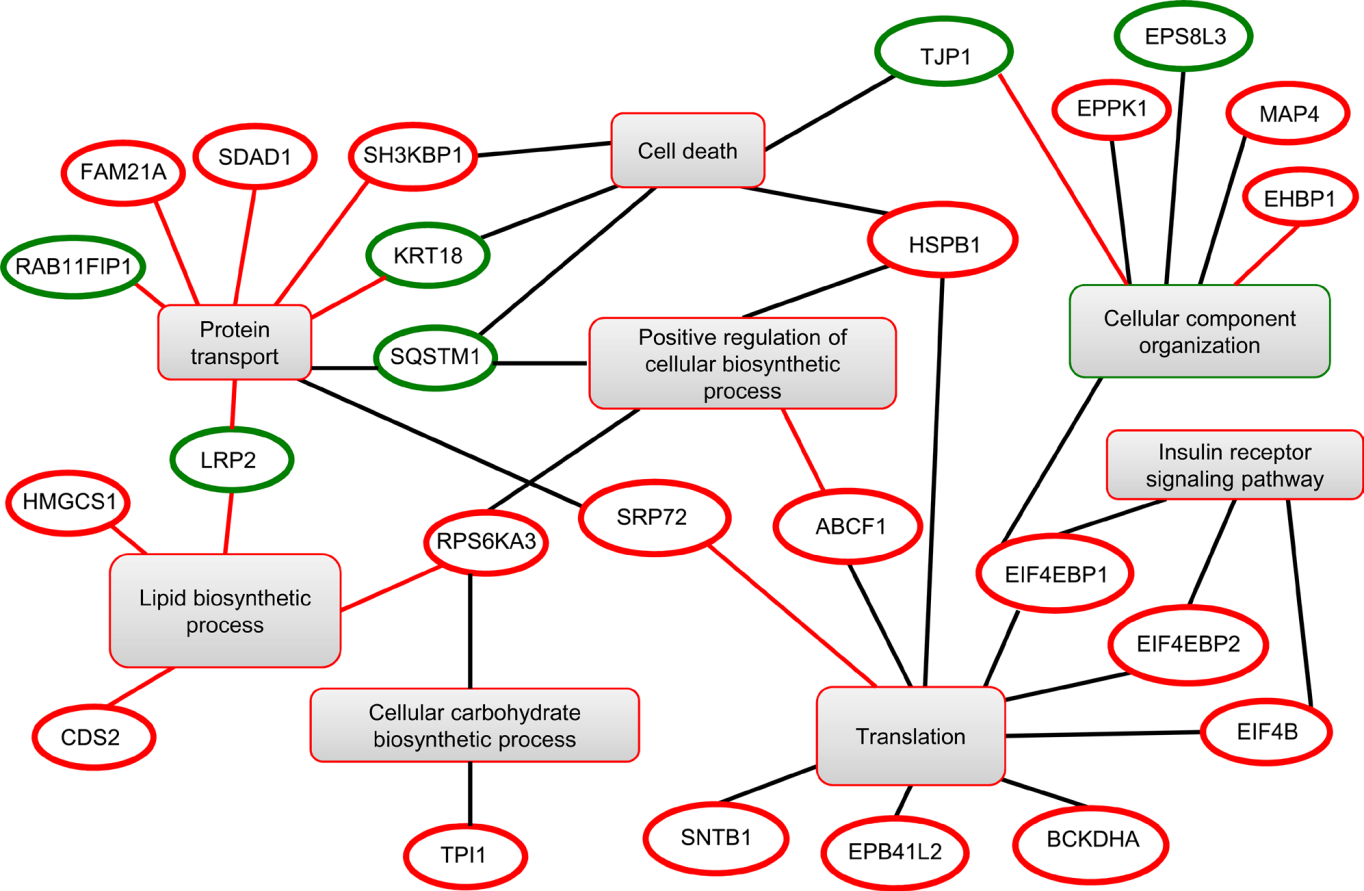
Functional clustering of the differentially modulated phosphoproteins identified by phosphoproteomics Red and green circles correspond to up- and down-phosphorylated proteins, respectively.

A SILAC-based phosphoproteomic approach was applied to unveil the signal transduction pathways modulated by HCV core protein. Hence, HuH7 and HuH7 expressing HCV core protein were cultured in SILAC media for metabolic labeling. Cells were lysed and the protein lysates were pooled in equimolar ratio prior to in-solution trypsin digestion. Phosphopeptides were further selectively enriched using an immobilized metal affinity chromatography (IMAC) resin and then analyzed by liquid chromatography coupled to tandem mass spectrometry (LC-MS/MS). The strategy exhibited the major advantage to allow simultaneously identification and accurate quantification of phosphorylated proteins under the different conditions. This state-of-the-art proteomic approach was applied to the cellular models in three independent SILAC experiments and led to the identification of 7 proteins down-phosphorylated and 24 proteins hyper-phosphorylated in HCV core expressing cells (Table 1). In addition, 16 out of the 35 differentially phosphorylated peptides exhibited a modulation of phosphorylation within the (Ser/Thr)-Pro motif, broadly recognized as potential MAP kinase and mTORC1 target sites [14], indicating that the (Ser/Thr)-Pro motif is essential for dynamic signaling transduction in HCV-core-expressing cells [15]. A functional network was built by investigating the functions of the identified proteins into Go Elite database and visualized in Cytoscape program. This approach revealed that the phosphoproteins modulated by HCV core were prominently involved in metabolism (carbohydrate and lipid biosynthetic processes), insulin receptor signaling, protein transport, cell death, cellular component organization and translation (Figure 1).

**Table 1:** Proteins and peptides with modulated phosphorylation in HuH7 cells expressing HCV core protein

						Ratio cT/ WT		
Gene Names	Accession number	Protein Names	Modified sequence	Probabilities of phosphorylation on STY	Phosphorylated position	S_1_	S_2_	S_3_
BCKDHA	F5H5P2	2-oxoisovalerate dehydrogenase subunit alpha, mitochondrial	IGHHS(ph)T(ph)SDDSSAYRSVDEVNYWDKQDHPISR	S(0.424)T(0.424)	S371/T372	2.58	_	_
			IGHHS(ph)TSDDSSAY(ph)RSVDEVNYWDK	S(0.748)Y(0.283)	S371/Y379	_	2.36	1.54
SNTB1	Q13884	Beta-1-syntrophin	LVHSGPGKGS(ph)PQAGVDLSFATR	S(1)	S389	3.8	1.67	1.83
SH3KBP1	Q96B97	SH3 domain-containing kinase-binding protein 1	S(ph)IEVENDFLPVEK	S(1)	S230	2.05	_	2.08
RPS6KA3;RPS6KA6	P51812	Ribosomal protein S6 kinase alpha-3	KAYS(ph)FCGTVEYMAPEVVNRR	S(0.987)	S227	_	2.57	_
			TPKDS(ph)PGIPPSANAHQLFR	S(0.998)	S369	1.5	3.98	1.77
SDAD1	Q9NVU7	Protein SDA1 homolog	YIEIDS(ph)DEEPRGELLSLR	S(1)	S585	2.11	1.7	1.5
FAM122A	Q96E09	Protein FAM122A	RIDFIPVS(ph)PAPS(ph)PTR	S(1)S(0.99)	S143/S147	_	2.03	1.59
EIF4EBP1	Q13541	Eukaryotic translation initiation factor 4E-binding protein 1	RVVLGDGVQLPPGDYSTT(ph)PGGTLFSTT(ph)PGGTR	T(0.968)T(0.493)	T37/T46	1.15	2.95	2.23
EIF4EBP2	Q13542	Eukaryotic translation initiation factor 4E-binding protein 2	TVAISDAAQLPHDYCTT(ph)PGGTLFSTT(ph)PGGTR	T(0.5)T(0.469)	T37/T46	_	1.96	1.81
SRP72	O76094	Signal recognition particle 72 kDa protein	TVSSPPTS(ph)PRPGS(ph)AATVSASTSNIIPPR	S(0.31)S(0.354)	S625/S630	_	2.03	1.81
EHBP1	Q8NDI1	EH domain-binding protein 1	DLSTS(ph)PKPSPIPS(ph)PVLGR	S(0.739)S(0.998)	S428/S436	1.67	_	2
			DLSTS(ph)PKPS(ph)PIPS(ph)PVLGR	S(0.401)S(1)S(1)	S428/S432/S436	1.52	2.29	2
TMPO	P42167	Lamina-associated polypeptide 2, isoforms beta/gamma	GPPDFS(ph)S(ph)DEEREPT(ph)PVLGSGAAAAGR	S(1)S(1)T(0.987)	S66/S67/T74	_	2.08	1.52
SEC16A	J3KNL6	Protein transport protein Sec16A	GSVSQPS(ph)T(ph)PS(ph)PPKPTGIFQTSANSSFEPVK	S(0.715)T(0.715)S(0.81)	S592/T593/S595	_	2.97	1.88
TPI1	P60174	Triosephosphate isomerase	KQS(ph)LGELIGTLNAAK	S(1)	S58	1.63	2.41	2.79
NUCKS1	Q9H1E3	Nuclear ubiquitous casein and cyclin-dependent kinase substrate 1	KVVDYS(ph)QFQESDDADEDYGRDSGPPTK	S(0.526)	S14	_	2.2	1.79
			VVDYSQFQES(ph)DDADEDYGRDSGPPTK	S(0.992)	S19	_	2.07	1.82
FAM21A;FAM21B	Q641Q2	WASH complex subunit FAM21A;WASH complex subunit FAM21B	ASALLFS(ph)S(ph)DEEDQWNIPASQTHLASDSR	S(0.999)S(1)	S619/S620	_	1.51	2.69
CDS2	O95674	Phosphatidate cytidylyltransferase 2	VAHEPVAPPEDKES(ph)ESEAKVDGET(ph)ASDSESR	S(0.714)T(0.537)	S21/T31	_	1.59	2.39
MAP4	E7EVA0	Microtubule-associated protein 4	DMES(ph)PTKLDVTLAK	S(0.995)	S297	_	2.27	2.2
EIF4B	E7EX17	Eukaryotic translation initiation factor 4B	SLENETLNKEEDCHSPT(ph)SKPPKPDQPLK	T(0.917)	T466	_	1.63	3.39
SYAP1	Q96A49	Synapse-associated protein 1	EQDLPLAEAVRPKT(ph)PPVVIK	T(1)	T248	_	2.37	1.52
HMGCS1	Q01581	Hydroxymethylglutaryl-CoA synthase, cytoplasmic	RPTPNDDTLDEGVGLVHSNIATEHIPS(ph)PAK	S(0.999)	S495	1.52	_	3.82
EPB41L2	O43491	Band 4.1-like protein 2	EVRS(ph)PTKAPHLQLIEGK	S(1)	S598	_	1.77	2.16
EPPK1	P58107	Epiplakin	RQVS(ph)ASELHTSGILGPETLR	S(0.918)	S2716	1.71	2	_
ABCF1	Q8NE71	ATP-binding cassette sub-family F member 1	KLS(ph)VPT(ph)S(ph)DEEDEVPAPKPR	S(1)T(1)S(1)	S105/T108/S109	2.69	2.12	_
HSPB1	P04792	Heat shock protein beta-1	GPS(ph)WDPFRDWYPHSR	S(1)	S15	4.21	3.02	2.99
TJP1	G3V1L9	Tight junction protein ZO-1	VQIPVSRPDPEPVS(ph)DNEEDSY(ph)DEEIHDPR	S(0.963)Y(0.783)	S125/Y132	0.54	0.55	_
HNF1B	P35680	Hepatocyte nuclear factor 1-beta	GRLS(ph)GDEGS(ph)EDGDDYDTPPILK	S(1)S(1)	S75/S80	0.39	0.38	_
EPS8L3	Q8TE67-3	Epidermal growth factor receptor kinase substrate 8-like protein 3	RS(ph)SS(ph)PEDPERDEEVLNHVLR	S(0.667)S(0.667)	S229/S231	0.46	0.4	_
LRP2	P98164	Low-density lipoprotein receptor-related protein 2	ES(ph)VAATPPPS(ph)PSLPAKPKPPSR	S(0.942)S(0.958)	S4608/S4616	0.2	0.26	_
RAB11FIP1	Q6WKZ4	Rab11 family-interacting protein 1	HLFSS(ph)TENLAAGSWKEPAEGGGLSSDR	S(0.805)	S357	0.31	0.46	_
KRT18	P05783	Keratin, type I cytoskeletal 18	STSFRGGMGS(ph)GGLATGIAGGLAGMGGIQNEK	S(0.776)	S60	_	0.32	0.55
SQSTM1	Q13501	Sequestosome-1	KIALESEGRPEEQMES(ph)DNCS(ph)GGDDDWTHLSSK	S(0.997)S(0.998)	S328/S332	_	0.54	0.41

The table encompasses the genes, protein names and their accession numbers according to the UniProt-SwissProt database. In the peptide sequences, (ph) after S/T/Y residue indicates the phosphorylated status. The probability for the site of phosphorylation was calculated by MaxQuant. The study was performed in biological triplicate (S_1_/S_2_/S_3_) and Phosphorylated peptides were considered to be significantly regulated when exhibiting an abundance ratio core/WT exceeding mean + 1.96 standard deviation in at least two replicates.

### HCV core protein enhances the level of phosphorylated 4E-BP1 in human hepatoma cells and primary hepatocytes

Among the proteins appearing substantially hyper-phosphorylated in HCV core protein expressing cells compared to the control, several components involved in translation were identified such as the eukaryotic translation initiation factor 4B (EIF4B) and the eukaryotic translation initiation factors 4E-binding protein 1 (4E-BP1) and 2 (4E-BP2). These proteins play a critical role in protein synthesis control [16] and their deregulation is associated with the development and progression of cancers because components of the translational machinery function at the point of convergence of deregulated cell signaling pathways.

**Figure 2:**
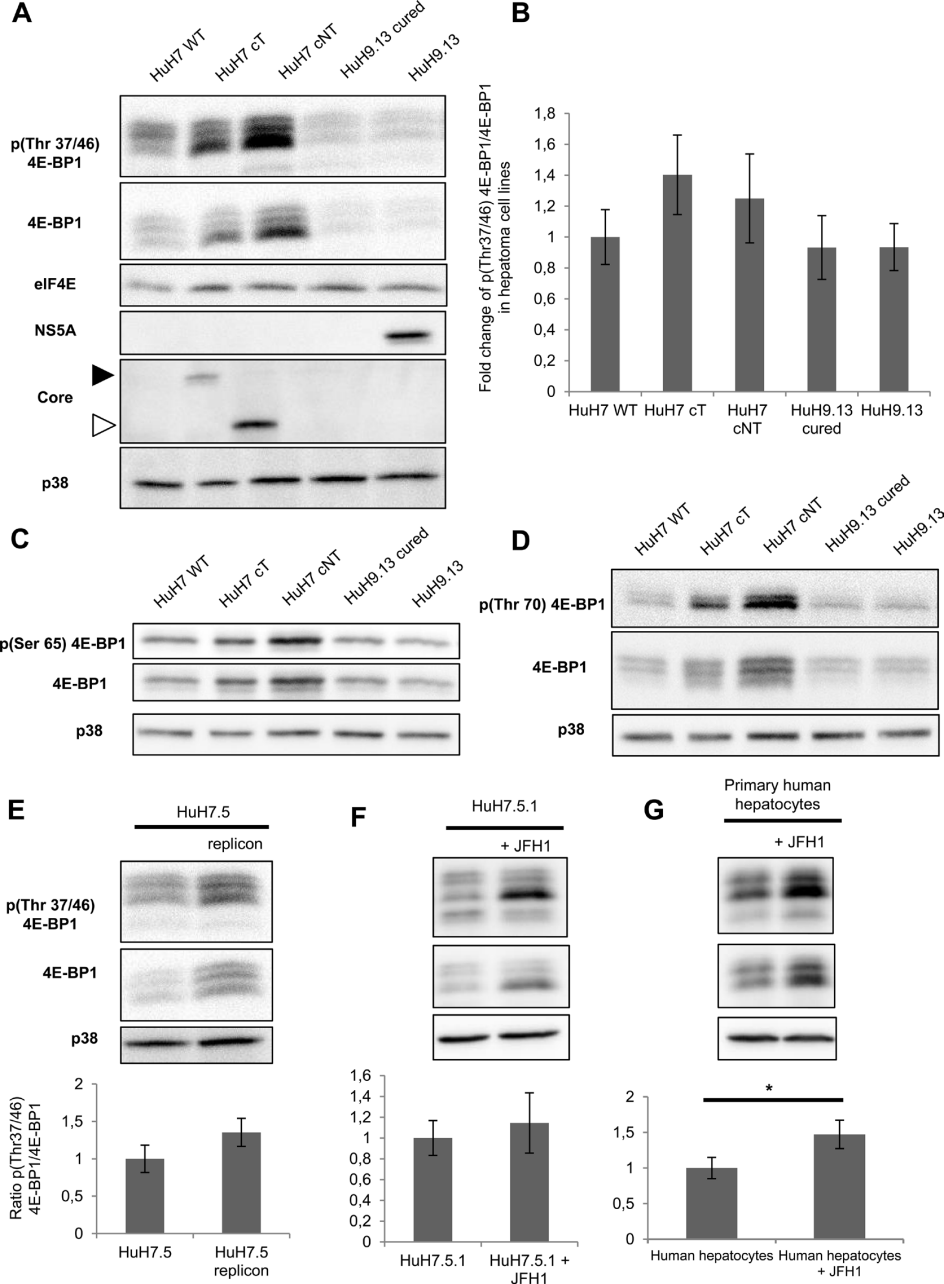
HCV core protein increases the level of phosphorylated 4E-BP1 (**A**) Immunoblotting analysis of 4E-BP1 phosphorylation on Thr 37/46 in lysates of HuH7 controls (WT), HuH7 stably expressing core protein variants either isolated from tumor or non-tumor cirrhotic (cT or cNT) areas and HuH9.13 harboring the HCV NS3-NS5B subgenomic replicon and its related control (HuH9.13 cured). HCV NS5A and core expressions are shown. When the membrane was probed with an antibody directed against core antibody, an expected signal at 20 kDa was obtained in HuH7 cNT (white arrow), however this signal was observed at 25 kDa in HuH7 cT since they express a FLAG-tagged core (black arrow). (**B**) Graphic representation of average fold changes of phospho Thr37/46 4E-BP1 normalized to 4E-BP1 (**C**, **D**) Protein lysates of HuH7 WT, cT, cNT, HuH9.13 cured and HuH9.13 were analyzed by Western blotting for the phosphorylation state of 4E-BP1 at Ser 65 and Thr 70, respectively. (**E**) Immunoblotting analysis of 4E-BP1 phosphorylation at Thr 37/46 and the relative ratios of p4E-BP1/4E-BP1 in HuH7.5 replicon, harboring all HCV proteins. (**F**, **G**) Protein extracts of HuH7.5.1 and primary human hepatocytes infected or not with the HCV strain JFH1 were immubloted with phospho Thr37/46 4E-BP1. Fold changes of p4E-BP1 over 4E-BP1 are shown, *p value < 0.05. One representative immunoblot out of three independent experiments is shown and p38 was used as loading control.

We further focused on 4E-BP1 phosphorylation because this event is critical in causing 4E-BP1 dissociation from eIF4E, which leaves 4E available to form the translationally active eIF4F complex and then results in switching on mRNA translation, which may contribute to changes in gene expression associated with malignant transformation [17]. To validate the phosphoproteomic findings and evaluate the state of 4E-BP1 phosphorylation on Thr 37/46, lysates prepared from HuH7 WT cells and HuH7 cells stably expressing core protein variants either isolated from tumor cT or non-tumor cirrhotic (cNT) areas, were analyzed by immunoblotting using an antibody that recognizes exclusively the dual phosphorylation on the residues Thr37/46. The amount of 4E-BP1 phosphorylated on Thr37 and Thr46 was significantly higher in HuH7 cT and cNT cells compared to HuH7 WT cells (Figure 2A and 2B), which matched and validated the aforementioned SILAC-based phosphoproteomics findings. The same increase in phosphorylated 4E-BP1 was observed with both core variants although the cNT was much more expressed than the cT (Figure 2B). In accordance with numerous previous studies, 4E-BP1 and its phosphorylated form are detected as multiple bands on gels in the vicinity of 20 kDa [18]. Moreover, an increased amount of phosphorylated 4E-BP1 was associated with an enhancement of 4E-BP1 expression in agreement with previously reported studies [19]. 4E-BP1 phosphorylation on Thr37/46 primes its subsequent phosphorylation on Ser65 and Thr70, decreasing its affinity for eIF4E, which allows eIF4G and associated factors to bind to eIF4E [17]. Therefore, these potential 4E-BP1 phosphorylation sites were investigated by immunoblotting using specific antibodies. As shown in Figure 2C and 2D, an increase of phosphorylated 4E-BP1 at Ser65 and Thr70 was observed in HuH7 cells expressing both HCV core cT and cNT compared to HuH7 WT.

To further substantiate the assumption of the HCV core protein-driven increase of phosphorylated 4E-BP1, human HuH9.13 cell line, harboring HCV NS3-NS5B subgenomic replicon, was herein used together with controls cured from the replicon. Interestingly but unexpectedly, HuH9.13 exhibited a drastically lower level of phospho-4E-BP1 on Thr37/46, Ser65 and Thr70 compared to HuH7 cT/cNT in immunoblot analysis (Figure 2A–2D). Whereas previous studies have shown that NS5A expressing cells (NS5A-HuH7.5) induce 4E-BP1 hyper-phosphorylation [20], a very weak signal was detected with the HuH9.13 used in this study, despite NS5A expression (Figure 2A–2D). Moreover, the increase in 4E-BP1 phosphorylation was also observed in HuH7.5 cells expressing the entire replicon (Figure 2E), confirming the efficacy of HCV core to promote 4E-BP1 phosphorylation in presence of all HCV proteins. Similarly, phospho-4E-BP1 levels were enhanced in JFH1-infected HuH7.5.1 in comparison with mock cells (Figure 2F). Notably, the enhancement in 4E-BP1 phosphorylation was likewise retrieved in primary human hepatocytes infected with JFH1 (Figure 2G). Strikingly fold changes of phospho-4EBP1 normalized to 4E-BP1 expression were not significantly different in hepatoma cell lines, however it turned significant in primary human hepatocytes (Figure 2B, 2G). This observation could be linked to the derivation of the HuH7 cell line from an HCC since it has been reported that 4E-BP1 phosphorylation was already increased in HCC tissues [19].

Taken together, these data experimentally confirmed the quantitative phosphoproteomic findings and strongly support the hypothesis that both HCV core variants cT and cNT induce 4E-BP1 dual phosphorylation in a hepatoma cell line. Importantly this effect was also observed in HCV-infected primary human hepatocytes.

### HCV core promotes 4E-BP1 phosphorylation *in vivo*

The above-mentioned *in vitro* observations raised the question of whether HCV core protein could drive an increase in the level of phosphorylated 4E-BP1 *in vivo*. Therefore, we used transgenic mice harboring two core variant sequences cT and cNT [4]. These mice did not exhibit demonstrable deficits in growth or development and no cellular inflammatory infiltrate was evident in their livers. Interestingly, when liver proteins extracted from these transgenic mice were analyzed for core by Western blot, a strong signal was detected at the expected 20 kDa in cNT, whereas a faint signal was visible in cT. To check the transcription level of HCV core, RNA was isolated from 3 different WT, cT and cNT transgenic mouse livers, reverse transcribed into cDNA, amplified and quantified by qPCR for HCV core gene. The same transcription level of HCV core messenger in cT and cNT mouse livers was detected (Supplementary Figure 1). Thus, the low protein level of HCV core cT could be related to its instability and its further degradation. This hypothesis was confirmed by a significant increase of cT protein expression in cells treated with MG132, a proteasome inhibitor, which effectively blocks the proteolytic activity of the 26S proteasome complex (Supplementary Figure 1).

**Figure 3:**
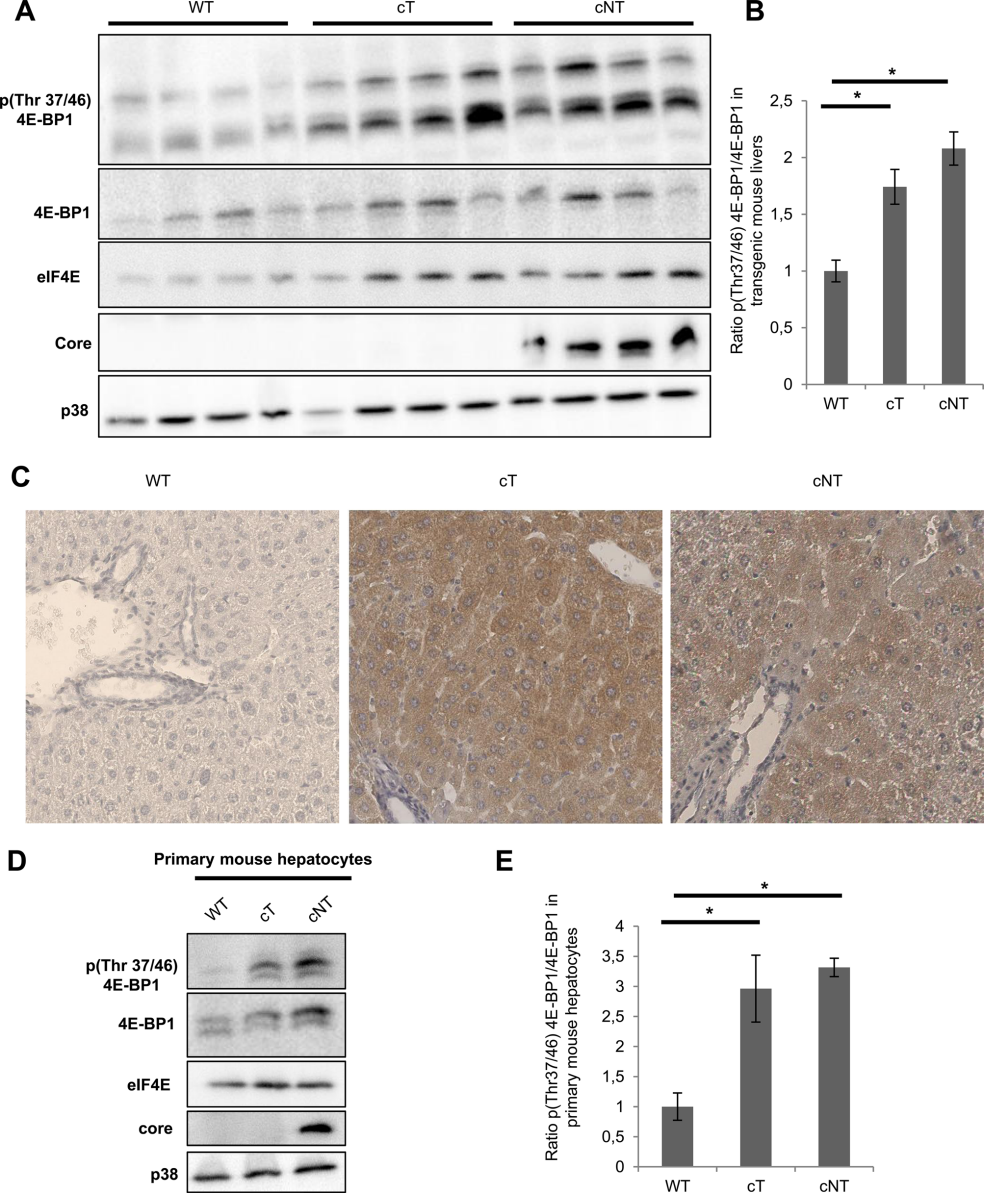
HCV core variants mediate 4E-BP1 phosphorylation in transgenic mouse livers (**A**) Liver proteins extracted from 9-month-old WT, transgenic mice that specifically express HCV core protein isolated from tumor and non-tumor (cT or cNT) areas (4 different mice per group) were analyzed for phospho 4E-BP1 by immunoblot. (**B**) Quantification of phospho-4E-BP1 relative to 4E-BP1 levels, *p value < 0.05. (**C**) Immunohistochemical staining of WT, cT and cNT liver biopsies. Liver slices were immunostained with p4E-BP1 antibody and representative results are shown (magnification x40). (**D**) Protein lysates of primary mouse hepatocytes isolated from transgenic mouse livers expressing or not cT and cNT were analyzed by immunoblot with antibodies directed against phospho 4E-BP1. One representative experiment is shown and p38 is used as loading control. (**E**) Depiction of normalized densitometric values of phospho-4E-BP1 over 4E-BP1 in primary mouse hepatocytes, *p value < 0.05.

To study the 4E-BP1 phosphorylation status in the liver of these transgenic animals, Western blot assays were performed on different 9-month-old mouse liver protein lysates and a representative example of protein extracts from four mice per group is shown in Figure 3. In agreement with *in vitro* data, immunoblotting of lysates from cT and cNT mouse liver showed a significant HCV core protein dependent increase of 4E-BP1 phosphorylation on Thr37/46 in comparison with the WT (Figure 3A and 3B). Of note, the observations made *in vivo* not only indicated an increased amount of phosphorylated 4E-BP1 at constant phosphorylation stoichiometry as it was the case *in vitro*; this time cT and cNT also enhanced the phosphorylation level of the protein 4E-BP1. Furthermore, immunohistochemical staining of p4E-BP1 was performed on transgenic mouse livers. Figure 3C shows the increase in p4E-BP1 in cT and cNT mouse livers compared to the WT. Next, it was relevant to explore the phosphorylation state of 4E-BP1 in primary cultured mouse hepatocytes isolated from WT and transgenic mouse livers expressing either HCV core protein variant cT or cNT. To this end, we compared the phosphorylation changes of 4E-BP1 by Western blot assays. Although the total 4E-BP1 expression was found to be enhanced in transgenic mouse livers a significant phosphorylation increase of 4E-BP1 in cT and cNT mouse hepatocytes was observed (Figure 3D and 3E). Shutdown of 4E-BP1 phosphorylation was observed in core primary mouse hepatocytes upon treatment with MEK/ERK and mTORC1 inhibitors, suggesting that these signaling pathways could be involved in the increase of 4E-BP1 phosphorylation by HCV core protein (Supplementary Figure 2).

Altogether these results corroborate the assumption that HCV, through core protein, enhances both total protein level and phosphorylation stoichiometry of 4E-BP1 in hepatocytes of transgenic mouse livers.

### HCV core induces the expression of MTA1, a 4E-BP1 downstream target

**Figure 4:**
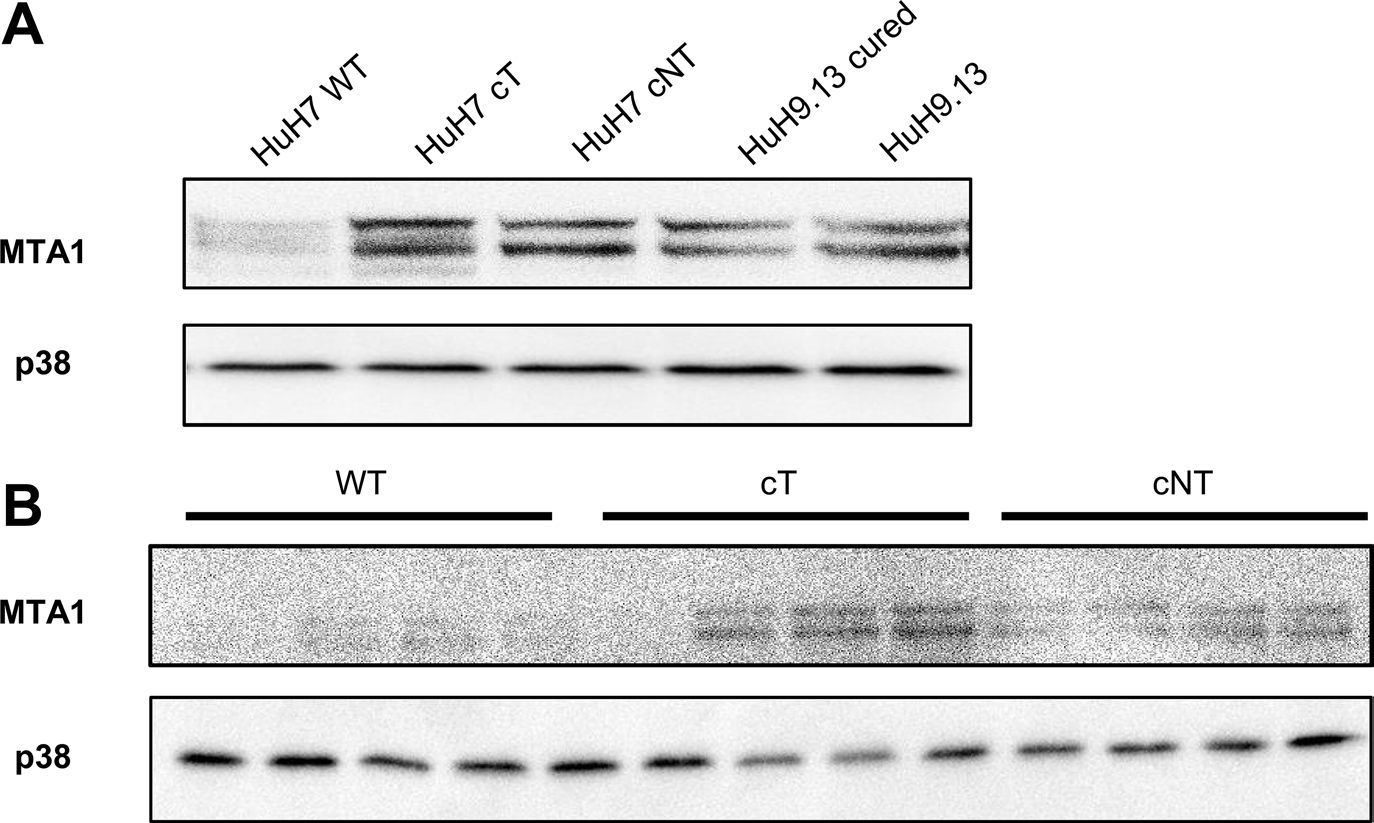
HCV core induces the expression of MTA1, a 4E-BP1 downstream target (**A**) Immunoblotting analysis of MTA1 expression in lysates of HuH7 controls (WT), HuH7 stably expressing core protein variants either isolated from tumor or non-tumor cirrhotic (cT or cNT) areas and HuH9.13 harboring the HCV NS3-NS5B subgenomic replicon and its related control (HuH9.13 cured). (**B**) Liver proteins extracted from 9-month-old WT, transgenic mice that specifically express HCV core protein isolated from tumor and non-tumor (cT or cNT) areas (4 different mice per group) were analyzed for MTA1 expression by immunoblot.

It has been reported that expression of a non phosphorylable 4E-BP1 mutant decreases YB1, vimentin, CD44 and MTA1 expressions at the protein but not mRNA level and thus could represent downstream targets of 4E-BP1 [21]. Among them, we chose to study MTA1 expression because this protein was reported to be highly expressed in HCC and served as an indicator of poor prognosis in HCC patients [22]. Our results show that increased expression of the phosphorylated forms of 4E-BP1induced by HCV core in HuH7 (Figure 4A) as well as in mouse livers was associated with a marked increase in MTA1expression (Figure 4B). Consistent with these findings we have previously reported that another gene of this 4E-BP1 signature, vimentin was upregulated in hepatocytes expressing HCV core [4]. These data provide evidence of downstream molecular effects of 4E-BP1 activation by HCV core.

### HCV core protein cooperates with Myc in hepatocarcinogenesis

Previous reports have delineated a significant link between c-Myc and 4E-BP1 phosphorylation in Myc-driven hematological cancers [23]. Since none of the core transgenic mice had developed malignant lesions, it was therefore relevant to uncover whether HCV core turns oncogenic in a cancer predisposed atmosphere.

**Figure 5:**
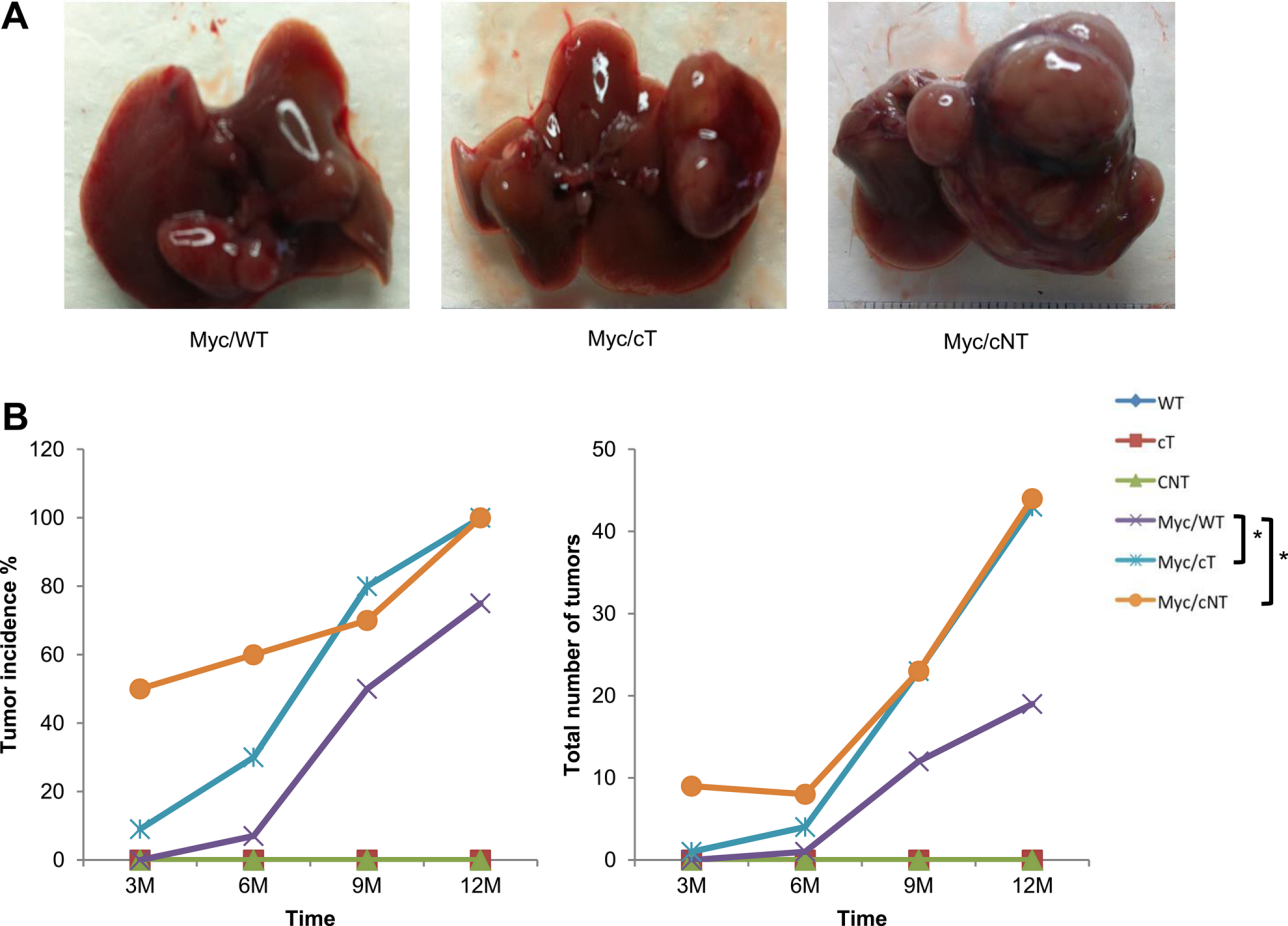
HCV core protein accelerates Myc-induced carcinogenesis in double transgenic mice (**A**) Gross images of Myc/WT, Myc/cT and Myc/cNT transgenic mouse livers. (**B**) Graphical representation of the percentage of tumor incidence and the total number of tumors in all transgenic mouse groups at 3, 6, 9 and 12 months, *p value < 0.05.

Hence, transgenic mice expressing either HCV core protein cT or cNT were intercrossed with Myc transgenic mouse lines in which liver-specific expression of c-Myc driven by woodchuck hepatitis virus (WHV) regulatory sequences causes liver cancer in all animals [24]. Liver tumors were detected earlier in bitransgenic animals carrying either cT or cNT core transgenes when compared to simple Myc littermates (Figure 5). This earlier detection was significant for Myc/cNT (*p* = 0.003) and Myc/cT (*p* = 0.045) as compared to Myc/WT. It was equivalent in Myc/cNT and Myc/cT mice at 9 and 12 months where all bitransgenic mice developed HCC, even though it was more pronounced in Myc/cNT in 3-month-old mice. Moreover the total number of tumors was statistically increased (*p* = 0.034) without interaction of time (*p* = 0.759) in both Myc/cT or Myc/cNT bitransgenic mice compared to Myc simple transgenic mice and no significant difference was observed between the 2 core variants (*p* = 0.3).

**Figure 6:**
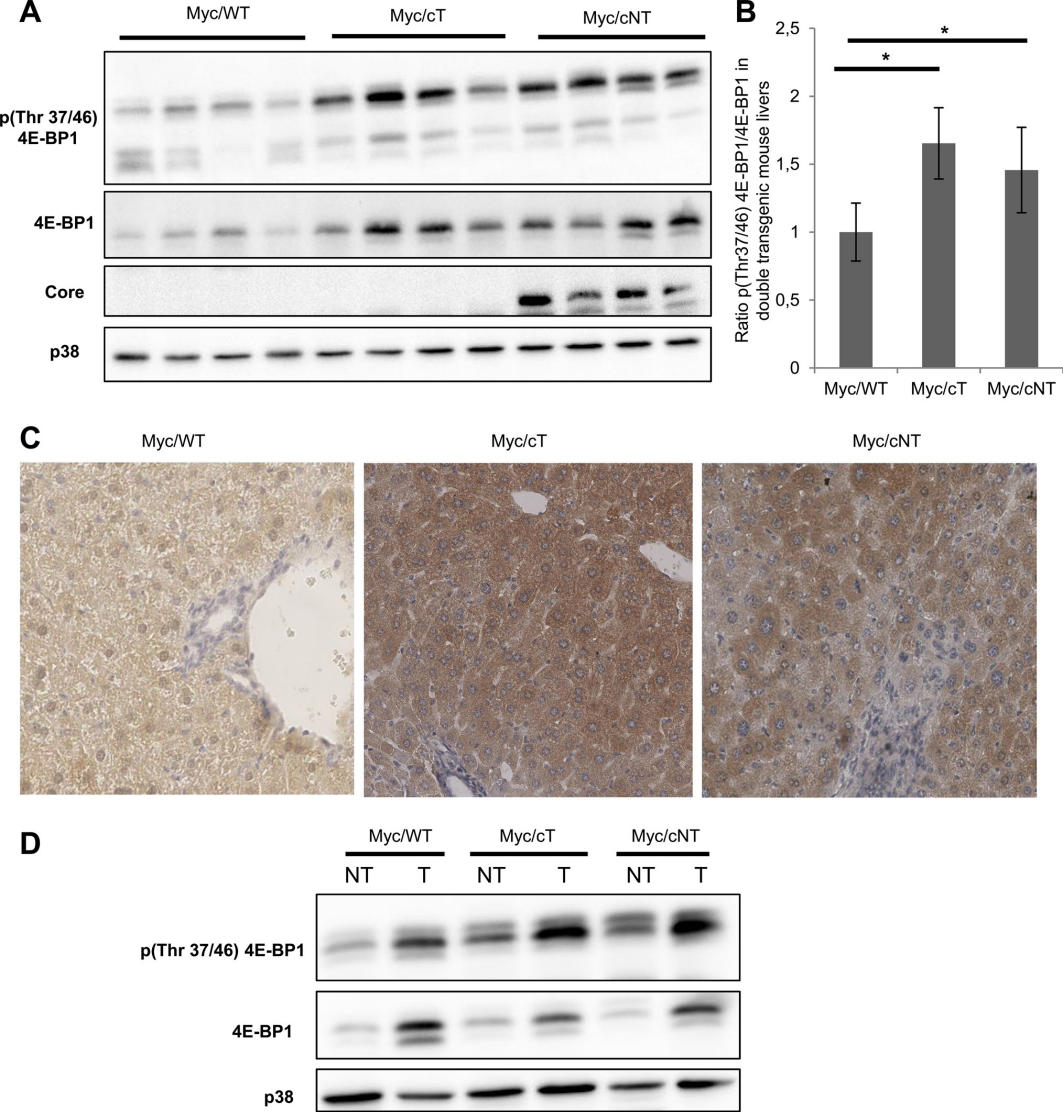
HCV core increases 4E-BP1 phosphorylation level in Myc/cT and Myc/cNT mouse livers (**A**) Representative immunoblotting assay of phospho 4E-BP1 on proteins extracted from livers of 3-month-old simple Myc/WT or double transgenic, Myc/cT and Myc/cNT mice (4 different mice/group). (**B**) Average fold changes of densitometric values of phospho Thr37/46 4E-BP1 expression relative to 4E-BP1 levels are represented graphically, *p value < 0.05. (**C**) Immunohistochemical analysis of tissue slices from paraffin-embedded livers of simple Myc/WT or double transgenic mice, Myc/cT and Myc/cNT, using phospho-4E-BP1 (magnification x40). (**D**) Protein extracts from double transgenic liver tumors (T) and the adjacent non-tumor tissues (NT) of 9-month-old Myc/WT, Myc/cT and Myc/cNT were analyzed for phospho-4E-BP1. One representative immunoblot of three independent experiments is shown, p38 is used as loading control.

To correlate the marked acceleration of the onset of hepatocarcinogenesis in double transgenic mice Myc/cT and Myc/cNT to 4E-BP1 status, further analysis of 4E-BP1 phosphorylation state in 3-month-old mouse liver extracts was performed by Western blotting. Figure 6A and 6B show a representative immunoblotting of 4 different mouse liver extracts per group and indicate that both HCV core variants triggered 4E-BP1 hyper-phosphorylation in double transgenic Myc/cT and Myc/cNT mouse livers compared to Myc/WT littermates. This finding was sustained by immunohistochemical analysis of paraffin-embedded double transgenic mouse livers using phospho-4E-BP1 (Thr37/46) (Figure 6C). A more pronounced increase in the phosphorylation level of 4E-BP1 was observed in double transgenic mice expressing both HCV core and Myc proteins suggesting that 4E-BP1 phosphorylation could be a possible effector of HCV core induced acceleration of HCC development.

Next, we sought to track 4E-BP1 phosphorylation in liver tumors, thus Western blotting assays were performed on liver extracts from double transgenic liver tumors (T) and the adjacent non-tumor tissues (NT). Since tumor detection appeared later in Myc/WT than in Myc/cT and Myc/cNT mice, liver extract from 9-month-old mice were used to compare 4E-BP1 phosphorylation in the 3 experimental conditions. In agreement with the immunoblotting results of the upper panels, Figure 6D shows that both variants of HCV core significantly induced 4E-BP1 phosphorylation in NT tissues of Myc/core mouse livers as compared with Myc/WT. Furthermore, 4E-BP1 phosphorylation was more enhanced in liver tumors of all double transgenic mice when compared with non-tumorous surrounding counterparts. This increased 4E-BP1 phosphorylation was also observed in the tumors of Myc/WT mice suggesting a role of this phosphorylation in the context of Myc-induced cancerogenesis as previously reported [23].

Altogether, the present data indicate that HCV core protein and Myc act synergistically to induce hepatic tumor development. This correlates with an enhancement of 4E-BP1 phosphorylation on Thr37 and Thr46 that might serve as a priming oncogenic event.

## DISCUSSION

HCV core protein orchestrates a complex and dynamic interaction network with cellular proteins involved in signal transduction, transcription, nucleic acid binding, apoptosis, cell cycle, cytoskeleton and kinase activity [25]. Herein, a phosphoproteomic study was conducted to decipher the signaling pathways activated by HCV core in hepatoma cells. Most of the identified phosphorylated proteins could be clustered in the above-mentioned processes such as lipid homeostasis, cytoskeleton dynamics and interestingly translation initiation machinery. Indeed, different phosphosites identified in this phosphoproteomic study (eIF4B, 4E-BP1, 4E-BP2) are parts of different eukaryotic initiation factors that govern translational machinery. 4E-BP1 represents a master regulator of protein synthesis control that is often deregulated in cancer cells. It was reported that activation of the 4E-BP1/eIF4E axis selectively stimulates the expression of malignancy-related mRNAs [26] and phosphorylation of 4E-BP1 enables cancer cell survival by enhancing protein synthesis capacity. Validation of phosphoproteomic data by immunoblotting indicated that indeed, phosphorylation levels of 4E-BP1 were increased in HuH7 cells, primary mouse hepatocytes expressing either HCV cT or cNT core proteins and in HuH7.5.1 cells and primary human hepatocytes infected with the HCV strain JFH1. The same results were obtained when total livers of HCV cT or cNT transgenic mice were examined indicating that HCV core influences the phosphorylation status of 4E-BP1 *in vivo*. Increased expression of MTA1 in HuH7 or mouse livers expressing HCV cores supports the notion that core-induced 4E-BP1 phosphorylation leads to activation of at least some of 4E-BP1downstream targets.

Both cT and cNT variants exhibit the same capacity to increase the phosphorylation of 4E-BP1. However, it is interesting to note that the core cT is less expressed than cNT in all cell lines tested and that the cT protein expression is barely detected *in vivo* reflecting the expression level which is observed in infected human tissues. This implies that a weak expression of the cT is as effective as the highly expressed cNT, underlying the importance of core genetic variability and expression in mediating downstream responses.

The important finding of this study lies in the significant potentiation of tumor development when HCV core is expressed in oncogenic context. Indeed, the onset and the higher number of tumors were observed in Myc/HCV core double transgenic mice when compared to single Myc littermates. Thus, cooperation with a cellular oncogene was required to reveal the oncogenicity of HCV core, since no malignancy was observed in our simple transgenic animal models. This novel function of HCV core acting as Myc tumor promoter in liver tumorigenesis is in accordance with the assumption that HCV core is a tumor accelerator in chemically induced HCC in DEN treated transgenic mice [27, 28].

Although many reports associated the 4E-BP1/eIF4E axis to the development of different cancers, 4E-BP1 phosphorylation in HCV core transgenic mice was not sufficient to initiate carcinogenesis and thus subsequent oncogenic alterations were required to drive oncogenesis. This is consistent with the obtained results in HCV core/Myc double transgenic mice. In accordance with our results, it was recently reported that activation of the 4E-BP1/eIF4E axis alone was not able to promote malignant transformation of murine hepatocytes, but that eIF4E cooperates with activated N-Ras to induce liver tumor development in mice [19]. Furthermore, in a Eμ-Myc transgenic mouse model in which constitutive overexpression of Myc in the B-cell compartment drives lymphomagenesis, it was reported that Myc overexpression results in 4E-BP1 hyperphosphorylation that was maintained during tumor progression and was required for cancer cell survival in Myc-dependent tumor initiation and maintenance [23]. Moreover the 4E-BP1/eIF4E axis was reported as a key event in Myc lymphomagenesis since 4E-BP1-dependent inhibition of eIF4E activity impedes Myc-driven lymphomas [29]. In our model, 4E-BP1 was found to be hardly phosphorylated in non-tumor tissues from Myc/WT mice, but this phosphorylation level was greatly increased in tumor tissue. This is consistent with the findings that in WHV/Myc transgenic mice, Myc is transiently overexpressed in the liver after birth, and it is re-expressed at tumor onset [24]. More importantly, phosphorylated 4E-BP1 abundance was observed in non-tumor tissue of Myc/cT and Myc/cNT double transgenic and this phosphorylation was more pronounced in the tumors. It must be noted that, although in these transgenic mice the ratio between p4E-BP1/4E-BP1 was statistically significant, the total forms of 4E-BP1 were increased as compared to control mouse livers. Thus, as 4E-BP1 has been reported to be upregulated in many cancers it is possible that the non phosphorylated form plays also a role in the development of Myc-induced HCC.

Altogether our data clearly establish that 4E-BP1 expression and phosphorylation are important targets for HCV core-related effects. Furthermore this study reinforces the notion that HCV core may potentiate oncogenes by acting as a cooperating partner, in particular in c-Myc-induced liver carcinogenesis.

## MATERIALS AND METHODS

### Reagents

Antibodies: 4E-BP1 (9452), phospho(Thr37/46) 4E-BP1 (2855), phospho(Ser65) 4E-BP1 (9451), phospho(Thr70) 4E-BP1 (9455), p44/42-MAPK (9102), phospho(Thr202/Tyr204) p44/42-MAPK (9101) and MTA1 (5647) were purchased from Cell Signaling, Core (sc-52403) and p38 (C-20, sc-535) provided from Santa Cruz. NS5A (ab13833) was from Abcam. Inhibitors: Rapamycin (9904) and PD0325901 (PZ0162) were purchased from Cell Signaling and Sigma, respectively.

### Transgenic mice

HCV core and WHV/c-Myc transgenic mice [24] were maintained in a pathogen-free facility, fed, and monitored in agreement with protocols approved by the guidelines of the Ministère de l’Agriculture (France). WHV/c-Myc transgenic mice, in which liver-specific expression of c-Myc is driven by woodchuck hepatitis virus (WHV) regulatory sequences, were previously described [24]. To establish HCV core transgenic mice, genotype 1b core cDNAs isolated from tumor (T) or cirrhotic nodules (NT) from the same patient were cloned downstream of hepatitis B virus regulatory element and microinjected into mouse embryos from the C57BL/6 (Institut Clinique de la Souris, Strasbourg, France). Both HCV core sequences, cT and cNT, were verified and correspond to previously published ones [3].

Homozygotes WHV/c-Myc transgenic mice were crossed with either homozygotes core T, core NT or C57BL/6 mice. The F1 were identified by hybridization of tail cDNA with WHV c-Myc and core probes. At least 10 mice per group were analyzed. Since it was previously shown that HCC development was more prominent in males, only males were selected in this study.

Double and single transgenic mice were euthanized at 3, 6, 9 or 12 months and livers were examined for tumor size and number. Tumor samples and adjacent liver tissues were fixed in 4% paraformaldehyde and embedded in paraffin for histopathological examination. For protein analysis, tumor samples were dissected free of adjacent liver tissues, capsule and necrosis, snap-frozen in liquid nitrogen and kept at −80°C. Livers from nontransgenic C57BL/6, mice were used as controls.

### Cell culture and viral infection

The hepatoma cell line HuH7 was obtained from ATCC. HuH7 cT and HuH7 cNT cells stably express HCV core protein variants isolated from tumor or non-tumor cirrhotic areas, respectively. HuH7.5 replicon cells express all HCV proteins. HuH9.13 cell line harbors the HCV NS3-NS5B subgenomic replicon. HuH9.13 cured cell line was obtained from HuH9.13 after 1month treatment with 500 U/ml interferon α2a as described [30]. HuH7.5.1 cells were gift from F.V. Chisari, the Scripps Research Institute, La Jolla, CA, USA [31]. All cell lines were cultured in Dulbecco's Modified Eagle's Medium (DMEM) supplemented with 10% fetal bovine serum and penicillin/streptomycin at 37°C in a humidified 5% CO_2_ atmosphere.

Primary hepatocytes of HCV core transgenic mice were isolated by *in situ* collagenase perfusion of livers as previously described [4].

Human primary hepatocytes freshly isolated from HCV-seronegative adult patients were purchased from Biopredic (Rennes, France) and maintained in primary culture at 37°C in a humidified 5% CO_2_ atmosphere as described previously [32]. Briefly, primary human hepatocytes were resuspended in complete medium consisting of Leibovitz's L-15 medium (Invitrogen, Cergy Pontoise, France) supplemented with 26 mM NaHCO_3_, 100 μg/ml streptomycin, 100 U/ml penicillin, 100 IU/l insulin (Novo Nordisk, Bagsvaerd, Denmark) and 10% heat-inactivated FCS, and seeded onto 6-well plates pre-coated with calf skin type I collagen (Sigma-Aldrich) at a density of 1.6 × 10^5^ viable cells/cm^2^. The medium was replaced 16 h later with fresh complete medium supplemented with 1 μM hydrocortisone hemisuccinate (SERB, Paris, France), and cells were left in this medium until HCV inoculation 2 days later. A high-titre stock of JFH1-HCV [33] was produced as described previously [32]. HuH7.5.1 cells and primary human hepatocytes were inoculated at a multiplicity of infection of 0.2 and 2, respectively. The culture medium was replaced with the inoculum diluted in the smallest volume of fresh medium sufficient for covering the cells. The inoculum was removed after a 4-hour incubation, then cells were washed 3 times with phosphate-buffered saline and maintained in their respective complete medium for 3 (HuH7.5.1 cells) or 6 (primary human hepatocytes) days before lysis for Western blot analysis.

### Stable isotope labelling with aminoacids in cell culture and phosphopeptide enrichment

Metabolic labelling was performed using the SILAC approach where HuH7 cells, or HuH7 cells stably expressing HCV cT protein were cultured either in light, medium or heavy SILAC media. Inversion of SILAC labeling allowed us to compare three independent experiments while avoiding possible labeling biases. Cells were cultured in DMEM supplemented with 10% dialyzed fetal bovine serum, 50 mg l-proline, and either l-arginine (Arg0) and l-lysine (Lys0) (light medium), or ^13^C_6_^14^N_4_-l-arginine (Arg6) and 4,4,5,5-D_4_-l-lysine (Lys4) (medium medium), or ^13^C_6_^15^N_4_-l-arginine (Arg10) and ^13^C_6_^15^N_2_-l-Lysine (Lys8) (heavy medium) (Thermo Scientific). Cells were maintained in culture for at least five doublings and the efficiency of labeling was confirmed by MS/MS analysis.

Whole cell protein extracts were obtained by lysing cells in a RIPA lysis buffer containing 50 mM Tris-HCl pH 7.4, 1% NP-40, 150 mM NaCl, 0.25% sodium deoxycholate, 0.5% SDS, Benzon nuclease (Novagen), a protease inhibitor cocktail and phosphatase inhibitors (Roche). Total cellular protein extracts were precipitated with TCA/acetone and then resuspended in 8M urea in 100 mM triethylammonium bicarbonate (TEAB) pH 8.5. A Bradford assay was performed to equalize protein concentrations for subsequent 1:1:1 ratio mixing of the three lysates. Mixed lysates were reduced by 10 mM Tris (2-carboxyethyl)phosphine (TCEP) for 1h at 37°C and then alkylated by 20 mM methylmethanethiosulfonate (MMTS) for 30 min at room temperature. After 7-fold dilution in 100 mM TEAB, proteins were digested overnight with sequencing grade trypsin (Promega) at an enzyme/protein ratio of 1/100 (w/w) at 37°C. The total peptide mixtures were then acidified to ∼pH 3 using acetic acid (AA), desalted on a C18 spin columns (Harvard Apparatus) and eluted in 60% ACN, 3% AA. This eluate was diluted 1:1 with water to reach a composition of 30% ACN, 1.5% AA, compatible with phosphopeptide enrichment. Desalted peptides were enriched for phosphopeptides by incubation with 10 uL of packed IMAC beads (Sigma-Aldrich) for 2h on a wheel rotating at 15 rpm. After washing the beads three times with 30% ACN, 1.5% AA, phosphopeptides were eluted from the IMAC resin using the alkaline buffer 400 mM NH_4_OH. Eluates were neutralized with 10% AA and dried by vacuum centrifugation [34]. Finally, phosphopeptides were resuspended in 5% ACN, 0.1% formic acid (FA) for subsequent analysis by LC-MS/MS.

### Liquid chromatography and mass spectrometry

Phosphopeptide separation was performed on an Ultimate 3000 nano LC system (Dionex) equipped with a C18 column (Acclaim PepMap C18, 75 μm id × 15 cm length, 3 μm particle size, 100 Å porosity, Dionex) and online connected to an LTQ Orbitrap XL mass spectrometer equipped with an ETD (Electron Transfer Dissociation) module (Thermo-Fisher Scientific). The mobile phases consisted of solvents A = 5% ACN (v/v), 0.1% FA (v/v) in water and B = 80% ACN (v/v), 0.1% FA (v/v) in water. Phosphopeptides were separated at a flow rate of 0.3 μL/min using a linear gradient of 60 min ramping from 0 to 50% of solvent B followed by an increase to 100% of solvent B in 10 min. The column was finally washed with 100% of solvent B for 10 min followed by re-equilibration with solvent A for 30 min.

Column eluent was sprayed into the MS instrument operated with a lock mass of 445.1200 for more accurate mass measurements in FTMS mode. Survey spectra were acquired over the mass range m/z 400–1400 at a resolution of 30,000 in the Orbitrap cell. In two separate runs, MS/MS data were acquired in data-dependent mode by selecting from the FTMS spectra the six most intense precursor ions for fragmentation by collision induced dissociation (CID) or electron transfer dissociation (ETD). Former target ions selected for MS^2^, within a mass window of +/− 5 ppm, were dynamically excluded for 45s. In CID fragmentation mode, Multistage Activation (MSA) was applied, while considering the possible neutral losses of 32.67, 49.00, 65.33 and 98.00 to account for singly and doubly phosphorylated peptides [35]. In CID, only species of charge states 2+ and 3+ were selected for fragmentation; in ETD, those of charge states 2+ and above were selected. For ETD, fluoranthene was used as the electron donor. A supplemental activation was systematically applied at 20% to increase fragmentation efficiency. Peptides were fragmented after accumulating ions to a target value of 2.10^5^, with a maximum injection time of 500 ms and using a charge state dependent reaction time (following the formula: RT = 100 ms × 2/z, with z the precursor charge state).

### Assigning peptide sequences to MS/MS spectra and relative quantification

Raw spectra were searched against the human SwissProt protein database (released on July 30th 2013) using the Andromeda search engine implemented in the MaxQuant software (version 1.3.0.5) that includes features for SILAC-based quantitation and phosphosite localization. The SILAC label modifications and methylthio (C) for cysteine residues were set as fixed modifications in the database search. Methionine oxidation and phosphorylation on S, T and Y residues were added as variable modifications. The precursor and fragment ion mass tolerances were 5 ppm and 0.6 Da, respectively. Up to two missed cleavages were allowed for trypsin digestion and the false discovery rate (FDR) was set to 1% for both peptide and protein identifications.

High precision SILAC-based quantitation of proteins was achieved by MaxQuant in a fully automatic way. Peaks are detected in each MS scan, peptide hills over the m/z-retention time are taken into account for intensity integrations and ratio calculation.

Phosphorylation site localization was based on PTM scores [36] that assign probabilities for each possible amino acid and then allow grouping the phosphosites into three categories. Probability scores greater or equal to 0.75 were considered to allow definite phosphosite localization, scores smaller than 0.75 and greater or equal to 0.5 corresponded to ambiguously localized phosphosites, whereas scores smaller than 0.5 were indicated non-localized phosphosites. This proteomic approach applied to the cellular models in three independent SILAC experiments led to the identification of 1308 phosphopeptides differing by their amino acid sequence corresponding to an overall of 977 different proteins. Phosphopeptides showing abundance ratios core/WT greater than 1.96 standard deviations distant from the mean in at least two out of the three biological experiments, were considered to be significantly hyper-phosphorylated.

### Western blot analysis

Protein extracts were subjected to SDS-PAGE gels and transferred onto nitrocellulose membranes. The blots were probed with different antibodies according to the manufacturer's instructions. Experiments were repeated three times and signal quantification was performed using Genetools software.

### Immunohistochemistry

Immunohistochemical staining on mouse liver slices was performed on 4% paraformaldehyde-fixed, paraffin-embedded tissues. Deparaffinized sections were incubated in 3% hydrogen peroxide for 20 minutes to quench the endogenous peroxidase. For antigen retrieval, slides were treated in citrate buffer for 10 minutes and incubated overnight with rabbit phospho(Thr37/46) 4E-BP1 at 1/1500 dilution in PBS, followed by an incubation with HRP-labeled secondary antibody (Dako Envision Systems) for 30 minutes. Immunoperoxidase staining was carried out with diaminobenzidine.

### Statistical analysis

The total number of tumors was analyzed by a Fisher test using a 2-way ANOVA: variance analysis integrated 2 factors (time and transgenic mouse group). Interaction between these 2 factors was excluded on the basis of Myc/WT control mice. LSD (post Hoc) test was performed after Fisher test to discriminate significance of each capsid group as compared to controls Myc/WT. Comparisons between two groups were performed with *t* test, (*) *p* value < 0.05.

### Quantitative RT-PCR

Frozen liver tissues were homogenized using Precellys (Ozyme) in cell-lysis grinding buffer and total RNA was isolated using RNAeasy kit (Qiagen), according to the manufacturer's instructions. Total RNAs were subjected to RNase-free DNase (Ambion) and converted into cDNA by using a Revert Aid Premium First Strand (Fermentas) and qPCR with Light Cycler Fast Start DNA green master (Roche Diagnostics). *HPRT* was used as housekeeping gene and relative quantification was based on the 2^−ΔΔCT^ method .

## SUPPLEMENTARY MATERIALS FIGURES


