# Supplementary material for: Hepatitis C virus core protein targets 4E-BP1 expression and phosphorylation and potentiates Myc-induced liver carcinogenesis in transgenic mice
Source: Oncotarget. 2017 Apr 20;8(34):56228–42. doi: 10.18632/oncotarget.17280 (PMC5593557; doi:10.18632/oncotarget.17280)
Supplement: Supplementary file 1 [file oncotarget-08-56228-s001.pdf]

Hepatitis C virus core protein targets 4E-BP1 expression and phosphorylation and potentiates Myc-induced liver carcinogenesis in transgenic mice

Supplementary Materials

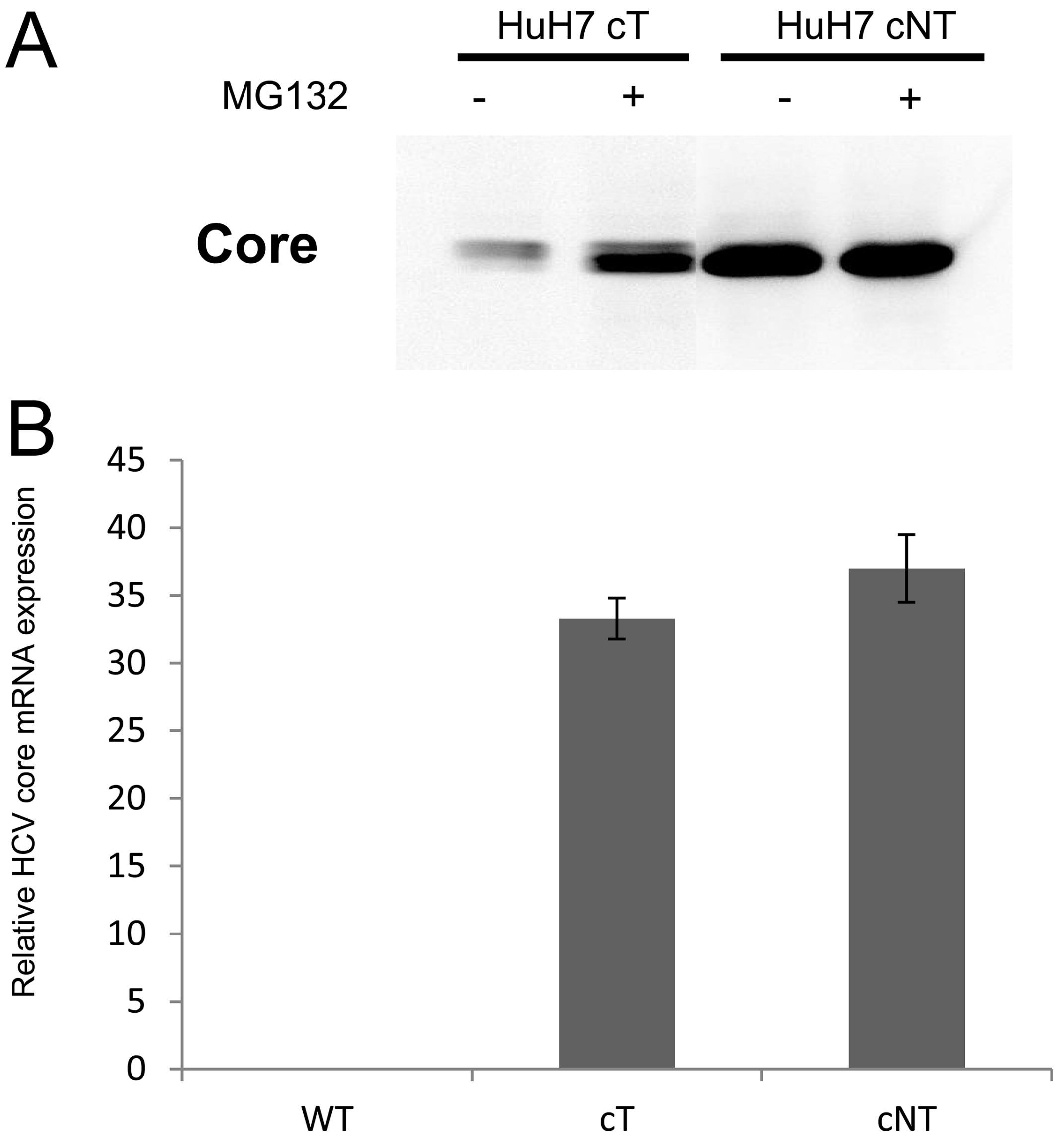

Supplementary Figure 1: (**A**) Increase of HCV core T expression by MG132 treatment. Protein lysates of HuH7 cT and cNT treated with the proteasome inhibitor MG132 (15 μM) were analyzed for core by western blot. (**B**) Relative HCV core mRNA levels of WT, cT and cNT transgenic mouse livers (3 mice per group).

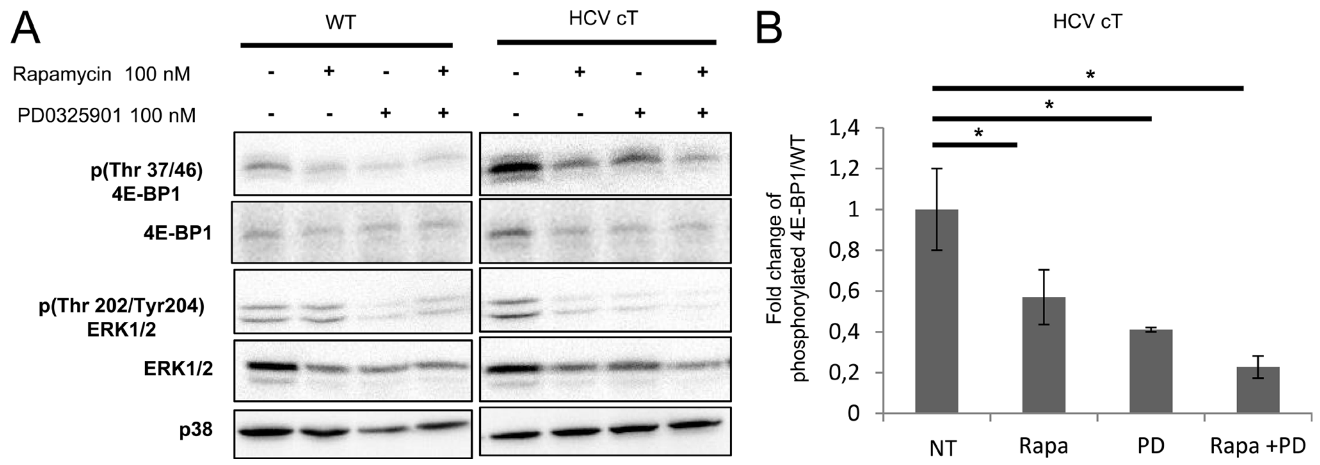

Supplementary Figure 2: Decrease of 4E-BP1 phosphorylation after rapamycin and PD0325901 treatments. (**A**) Primary mouse hepatocytes isolated from WT and HCV cT transgenic mice were treated with the mTOR1 inhibitor rapamycin (Rapa) and the ERK inhibitor PD0325901 (PD) either alone or in combination for 48 h. Cell lysates were immunoblotted with the indicated antibodies. (**B**) Graphic representation of average fold changes in p4E-BP1 in cT hepatocytes normalized to non-treated cells (NT) of three independent experiments, **p* value < 0.05. The same results were obtained when hepatocytes from cNT mice were used.
